# Calpain‐2 plays a pivotal role in the inhibitory effects of propofol against TNF‐α‐induced autophagy in mouse hippocampal neurons

**DOI:** 10.1111/jcmm.15577

**Published:** 2020-07-06

**Authors:** Ying Li, Zhiyong He, Hu Lv, Wei Chen, Jiawei Chen

**Affiliations:** ^1^ Department of Anesthesiology Fudan University Shanghai Cancer Center Shanghai China; ^2^ Department of Oncology Shanghai Medical College Fudan University Shanghai China

**Keywords:** autophagy, calcium, calpain, hippocampal neuron, inflammation, propofol

## Abstract

Calpains are calcium‐dependent proteases and play critical roles in neuronal autophagy induced by inflammation. Propofol has been reported to exert anti‐inflammatory effects in neurons. We aimed to identify whether and how propofol‐modulated calpain activity and neuron autophagy in response to tumour necrosis factor‐α (TNF‐α). Mouse hippocampal neurons were pre‐treated with propofol and exposed to TNF‐α. Autophagy was evaluated by fluorescent autophagy assay and by measuring LC3I and LC3II expression. Intracellular calcium concentration was measured by fluorescent assay. Calpain activation was measured by calpain activity assay. The protein expression of intracellular signalling molecules was detected by Western blot analysis. Compared with untreated control neurons, 40 ng/mL TNF‐α treatment for 2 hours induced neuron autophagy, which was attenuated by 25 μmol/L propofol. TNF‐α induced intracellular calcium accumulation, the phosphorylation of calcium/calmodulin‐dependent protein kinase II (CAMK II) and calpain‐2, calpain activation and lysosomal cathepsin B release as well as tyrosine kinase receptor B (TrkB) truncation. These effects were alleviated by propofol, calcium chelator, CAMK II inhibitor, calpain‐2 inhibitor, calpain‐2 siRNA transfection and N‐Methyl‐d‐aspartic acid (NMDA) receptor antagonist. Propofol, via NMDA receptor, inhibited TNF‐α‐mediated hippocampal neuron autophagy. The mechanism may involve calcium and calcium‐dependent signalling pathway, especially CAMK II and calpain‐2.

## 1 INTRODUCTION

Post‐operative cognitive dysfunction (POCD) is a clinical syndrome characterized by cognitive impairments in patients after anaesthesia and surgery,^1^ and its pathogenesis and pathophysiology remain inconclusive. In the past decade although multiple hypotheses, such as neuroinflammation, oxidative stress, mitochondrial dysfunction, blood‐brain barrier (BBB) impairment, synaptic dysfunction, lacking neurotrophic support and cholinergic failure,^2^ have been raised, neuroinflammation is believed to play an initial and central role in the development of POCD.^3^, ^4^, ^5^

During perioperative periods, pro‐inflammatory cytokines, such as tumour necrosis factor‐α (TNF‐α) and interleukin I, were released from activated macrophages and monocytes.^6^, ^7^, ^8^ The resultant neuroinflammation may lead to POCD through multiple pathways. It is well established that the hippocampal neurons, which play a central role in learning and memory, are particularly vulnerable to inflammatory insult due to its high density of receptors for inflammatory cytokines. We have reported that TNF‐α may cause mouse hippocampal neuron apoptosis.^9^ TNF‐α has been shown to modulate the electrophysiological properties of hippocampal neurons. Precisely speaking, it decreased after‐hyperpolarization amplitudes and learning ability in rats.^10^ Besides affecting hippocampal neurons, TNF‐α may also damage the component of BBB, potentially increasing its permeability and leading to brain injuries. It was reported that TNF‐α may alter the expression of matrix metalloprotein‐9 (MMP‐9) and occludin in cerebral microvascular endothelial cells,^11^, ^12^ thus increasing BBB permeability. In addition, TNF‐α could modulate synaptic plasticity, which is of considerable relevance to brain function.^13^ Recently, the role of hippocampal neuron autophagy in inflammation‐mediated neurodegenerative diseases is of great interests.

Autophagy is seen in all eukaryotic systems and is a natural and destructive mechanism that disassembles unnecessary or dysfunctional components. Thus, it enables cells to survive from the external environment stress like nutrient deprivation and also allows them to withstand internal stresses like accumulation of damaged organelles and pathogen. Although the activation of autophagy may be a protective mechanism against insults, dysfunctional or extensive autophagic activation is acknowledged as one of the features in neurodegenerative disorders such as Alzheimer's disease^14^ and traumatic brain injury‐induced cognitive impairments^15^ as well as POCD.^16^ Among multiple mechanisms for autophagy, calcium and calcium‐dependent calpains play critical roles.^17^, ^18^

Propofol is a widely used general anaesthetic, and its anti‐inflammatory and neuroprotective effects have been extensively investigated. Animal studies carried out in rats indicated that propofol may reduce inflammation‐induced brain injury,^19^ by inhibiting excessive activation of microglia^20^ and by suppressing inflammasome activation as well as pro‐inflammatory cytokines maturation.^21^ In vitro studies identified propofol could inhibit inflammation in neuroblastoma cells and BV2 microglia.^22^, ^23^ We previously reported that propofol may protect against TNF‐α‐induced hippocampal neuron apoptosis^9^ and could attenuate TNF‐α‐modulated expression of MMP‐9 and occludin in cerebral microvascular endothelial cells, thus maintaining BBB permeability.^11^, ^12^ Propofol also inhibited neuron autophagy,^24^ and further investigation revealed the involvement of calcium and calpains.^25^

In this study, we aimed to detect whether propofol and TNF‐α‐modulated hippocampal neuron autophagy, and further investigated the underlying mechanisms, which may involve calcium and related pathway.

## 2 MATERIALS AND METHODS

### 2.1 Experimental design

Primary mouse hippocampal neurons were cultured in neurobasal plus medium, exposed to different concentrations of TNF‐α (10, 20, 40, 80 ng/mL) for different times (1, 2, 4, 8 hours), and the extent of autophagy was examined. TNF‐α treatment condition that induced maximal autophagy was identified. To examine the effect of propofol, neurons were pre‐treated with different concentrations of propofol (1, 5, 10, 25, 50 μmol/L) or its solvent, 0.1% dimethyl sulfoxide (DMSO), for 1 hour, followed by TNF‐α treatment. We intended to identify the minimally effective concentration of propofol against TNF‐α‐induced autophagy. Then, the potential underlying mechanisms were investigated. In the present study, we focused on calcium and calcium‐dependent signalling pathway. TNF‐α and propofol were purchased from Sigma‐Aldrich. Calcium chelator, calcium/calmodulin‐dependent protein kinase II (CAMK II) inhibitor, calpain inhibitors and N‐Methyl‐d‐aspartic acid (NMDA) receptor antagonist as well as activator were purchased from Med Chem Express, and their concentrations in this study were suggested by previous publications^11^, ^23^, ^26^, ^27^ and confirmed to be effective in our preliminary experiments.

### 2.2 Cell culture

Primary mouse hippocampal neurons were purchased from Thermo Fisher Scientific and cultured in neurobasal plus medium (Thermo Fisher Scientific) supplemented with 25 µmol/L l‐glutamate, 5% foetal bovine serum, 100 units/mL penicillin and 100 μg/mL streptomycin. Neurons were incubated at 37°C in a humidified atmosphere with 5% CO_2_. The cryopreserved neurons were thawed and seeded into a tissue culture flask containing 5 mL medium, which was replaced every 2‐3 days. The cells were sub‐cultured when reaching 80%‐90% confluency, and the 4th passage of neurons was used in the present study.

### 2.3 Protein preparation

For whole‐cell extracts, after respective treatment, neurons were washed with phosphate buffer saline (PBS), scraped off and transferred to centrifuge tube. After centrifugation for 5 minutes at 268 *g*, pellets were suspended in ice‐cold RIPA lysis buffer containing protease inhibitor and phosphatase inhibitor for 5 minutes, followed by vortexing for 30 seconds. The proteins were obtained by centrifuging for 5 minutes at 1006 *g*. Cytoplasmic extracts were prepared with Cytoplasmic Extraction Reagents (Thermo Fisher Scientific) according to the manufacturer's instructions. Lysosomal extract was prepared with Lysosomal Extraction Kit (BioLab Technology) according to the manufacturer's instructions. Protein concentration was determined by BCA assay kit (Sigma‐Aldrich).

### 2.4 Protein measurement by Western blot analysis

After denaturing samples by heating at 95°C for 5 minutes, equal amounts of protein (about 50 μg/well) were subjected to 6%‐8% SDS‐PAGE and electrophoretically transferred to polyvinylidinene fluoride membranes (Sigma‐Aldrich). After being incubated in 5% non‐fat milk for 1 hour at room temperature, membranes were probed with specific primary antibody at 4°C for overnight with gentle rocking. Primary antibodies were obtained from Santa Cruz Biotechnology and Cell Signaling Technology and included antibody against calpain‐1, calpain‐2, phosphorylated calpain‐1, phosphorylated calpain‐2, CAMK II, phosphorylated CAMK II, cathepsin B, full‐length tyrosine kinase receptor B (TrkB), truncated TrkB, LC3I, LC3II and GAPDH. Then, the membranes were washed with Tris‐buffered saline with Tween (TBST) and incubated with corresponding secondary antibody (Santa Cruz Biotechnology) at room temperature for 1 hour. After being washed with TBST for 30 minutes, membranes were exposed to Amersham ECL plus Western blotting detection reagent (Santa Cruz Biotechnology), and protein visualization was performed with a chemiluminescent imaging system and analysed with Gel‐IT analysis software.

### 2.5 Construction and transient transfection of siRNA

The specific small interfering RNA (siRNA) against mouse calpain‐1 and calpain‐2 was designed and synthesized by Gene Pharma. The sequences of siRNA against calpain‐1 were 5′‐TAC CTC TGT TCA ATT GCT CTA‐3′ and 5′‐AAG CTA GTG TTC GTG CAC TCT‐3′, the sequences of siRNA against calpain‐2 were 5′‐GCG GTC AGA TAC CTT CAT CAA‐3′ and 5′‐AAA CCA GAG CTT CCA GGA AAA‐3′, and sequence of scramble control siRNA was 5′‐AAC GTA CGC GGA ATA CTT CGA‐3′. siRNAs were transfected with the lipofectamine 2000 reagent (Thermo Fisher Scientific) according to the manufacturer's instructions. Briefly, lipofectamine 2000 and siRNAs were mixed for 20 minutes at room temperature, followed by incubation with neurons. After culturing for 6 hours, neurons were washed and cultured in fresh media for 24 hours and exposed to treatment. The transfection efficiency was examined by measuring protein expression of target genes via Western blot analysis.

### 2.6 Measurement of intracellular calcium concentration

Intracellular calcium concentration was detected by calcium‐sensitive fluorescent indicator Fluo‐3 AM (Beyotime Biotechnology). In brief, after respective treatment, neurons were harvested, washed with PBS and suspended in 10 μmol/L Fluo‐3 AM for 45 minutes at 37°C in the dark room, followed by washing with PBS. Fluo‐3 AM fluorescence was excited at 488 nm, and the emission at 530 nm was obtained by flow cytometry. Data were expressed as fluorescent intensity.

### 2.7 Measurement of calpain activity

Calpain activity was determined with Calpain Activity Assay Kit from Abcam Inc according to the manufacturer's protocol. Fluorescence was measured using a Tecan Spectrafluor Plus fluorescent reader (Tecan) with wavelengths of 320 nm excitation and 480 nm emission at 30°C for 30 minutes. The initial velocity of the rate of increase in fluorescence was determined by calculating the slope after subtracting the background fluorescence. Data were expressed as fluorescent intensity.

### 2.8 Detection of autophagy

Neuron autophagy was detected with Autophagy Assay Kit from Abcam Inc according to the manufacturer's protocol. In brief, after treatment, neurons were collected, washed with assay buffer, suspended in green stain solution for 30 minutes at room temperature. The fluorescence was analysed with flow cytometry using a 488 nm laser source. Data were expressed as increased folds of fluorescent intensity in comparison with control neurons.

### 2.9 Statistical analysis

Data were presented as mean ± standard deviation. Differences between groups were assessed with Paired Student's *t* test, Student's Newman‐Keuls test (*q* test), one‐way ANOVA and Tukey's post hoc test. All statistical analyses were performed with SPSS software 11.5, and a significant difference was considered as *P* < .05. Sample size (n) represents the times of repeated experiments which were performed with different cultures.

## 3 RESULTS

### 3.1 TNF‐α time‐ and concentration‐dependently induced hippocampal neuron autophagy

**Figure 1:**
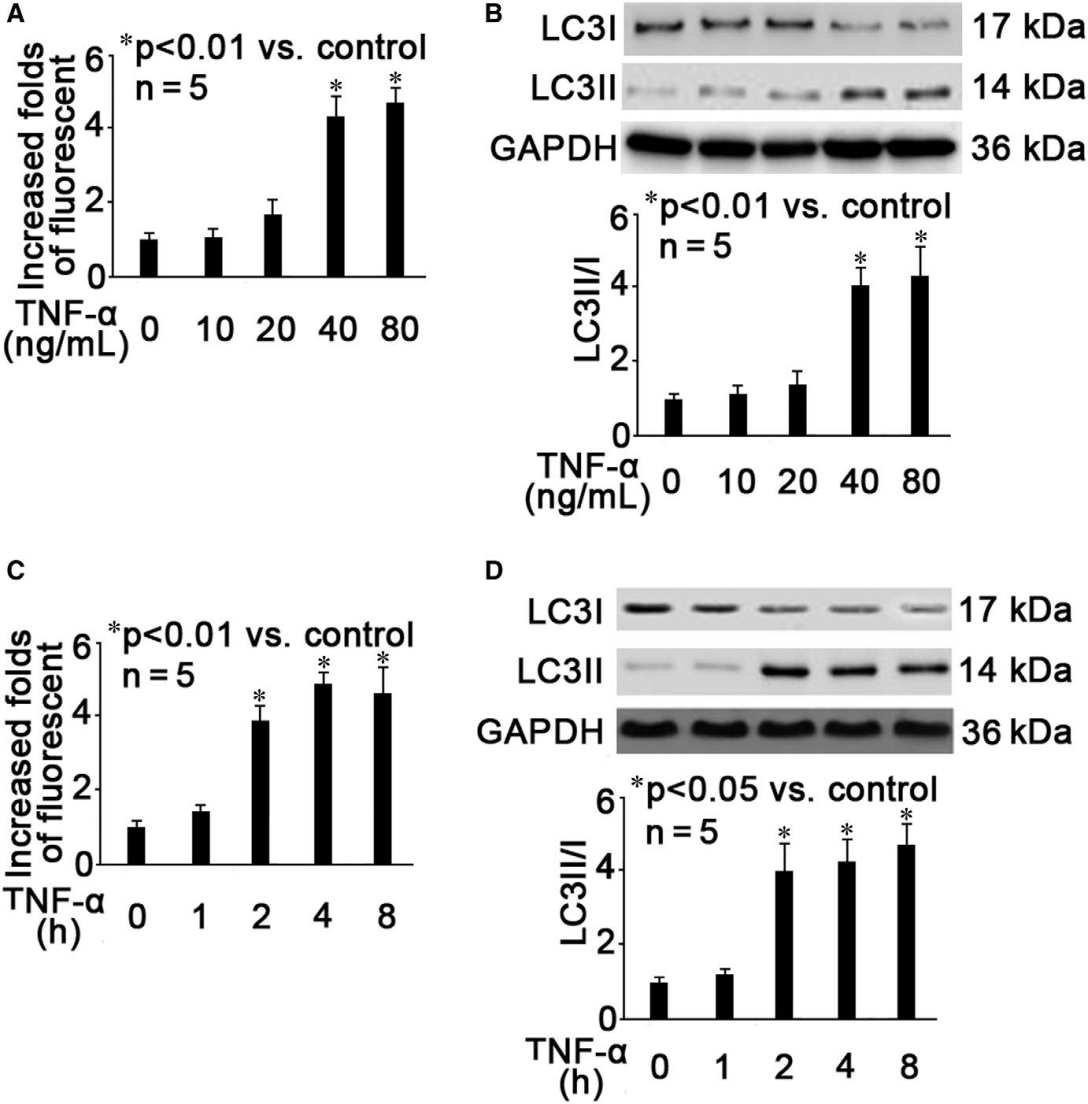
TNF‐α time‐ and concentration‐dependently induced hippocampal neuron autophagy. A, Neurons were treated with different concentrations (10, 20, 40, 80 ng/mL) of TNF‐α for 2 h, and autophagy was measured by flow cytometry. The fluorescent intensity of each group was recorded. Data were summarized from 5 separate experiments and were presented as increased folds of fluorescent intensity in comparison to control neurons. B, Neurons were treated with different concentrations (10, 20, 40, 80 ng/mL) of TNF‐α for 2 h, and autophagy was evaluated by measuring LC3I and LC3II expression as well as LC3II/I ratio. The upper panel was a representative experiment and the lower panel was the summary of data of LC3II/I expression ratio from 5 separate experiments. GAPDH served as loading control. Data were expressed as normalized ratio of protein band density of LC3II against LC3I, which was normalized with GAPDH. C, Neurons were treated with 40 ng/mL TNF‐α for different times (1, 2, 4, 8 h), and autophagy was measured by flow cytometry. The fluorescent intensity of each group was recorded. Data were summarized from 5 separate experiments and were presented as increased folds of fluorescent intensity in comparison to control neurons. D, Neurons were treated with 40 ng/mL TNF‐α for different times (1, 2, 4, 8 h), and autophagy was evaluated by measuring LC3I and LC3II expression as well as LC3II/I ratio. The upper panel was a representative experiment and the lower panel was the summary of data of LC3II/I expression ratio from 5 separate experiments. GAPDH served as loading control. Data were expressed as normalized ratio of protein band density of LC3II against LC3I, which was normalized with GAPDH

We treated hippocampal neurons with different concentrations (10, 20, 40, 80 ng/mL) of TNF‐α for 2 hours, and examined the extent of autophagy with autophagy assay kit. As shown in Figure 1A, we found that compared with untreated control neurons, 40 and 80 ng/mL TNF‐α treatment greatly induced autophagy (*P* < .01). TNF‐α‐induced autophagy was confirmed by measuring the ratio of LC3II/I, which is well accepted as a marker for autophagy. Consistently, we demonstrated that 40 and 80 ng/mL TNF‐α decreased the expression of LC3I and increased the expression of LC3II, resulting in increased ratio of LC3II/I (Figure 1B, *P* < .01 vs control neurons). In another set of experiment, we treated hippocampal neurons with 40 ng/mL TNF‐α for different times (1, 2, 4, 8 hours) and found that TNF‐α treatment for more than 2 hours induced autophagy significantly (Figure 1C, *P* < .01 vs control neurons). Also, 40 ng/mL TNF‐α treatment for more than 2 hours decreased the expression of LC3I and increased the expression of LC3II as well as LC3II/I ratio (Figure 1D, *P* < .05 vs control neurons). Accordingly, 40 ng/mL TNF‐α treatment for 2 hours was considered the optimal condition to induce hippocampal neuron autophagy.

### 3.2 Propofol concentration‐dependently alleviated TNF‐α‐induced hippocampal neuron autophagy

**Figure 2:**
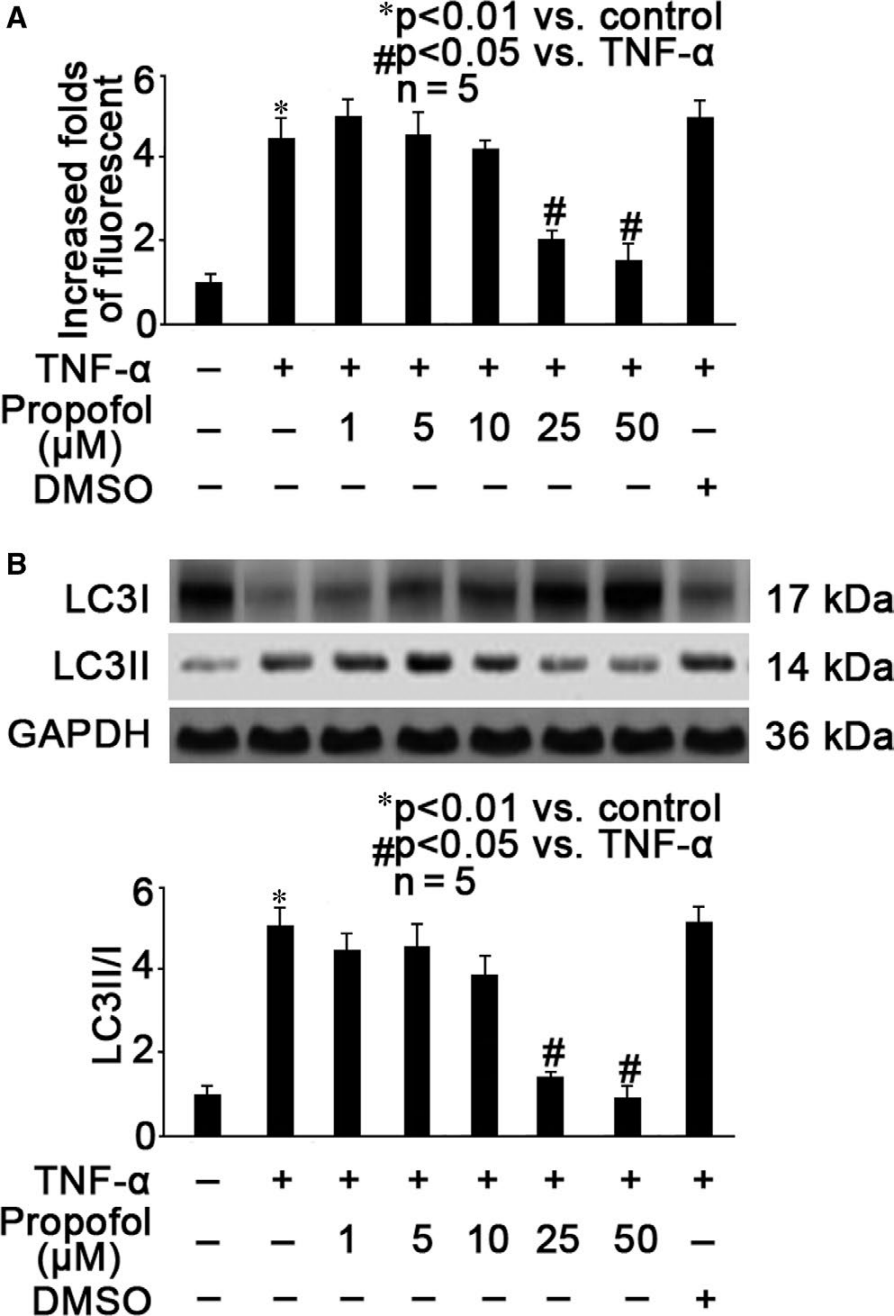
Propofol concentration‐dependently alleviated TNF‐α‐induced hippocampal neuron autophagy. A, Neurons were pre‐treated with different concentrations (1, 5, 10, 15, 50 μmol/L) of propofol or 0.1% DMSO for 1 hour, followed by TNF‐α treatment (40 ng/mL, 2 h), and autophagy was measured by flow cytometry. The fluorescent intensity of each group was recorded. Data were summarized from 5 separate experiments and were presented as increased folds of fluorescent intensity in comparison to control neurons. B, Neurons were pre‐treated with different concentrations (1, 5, 10, 15, 50 μmol/L) of propofol or 0.1% DMSO for 1 h, followed by TNF‐α treatment (40 ng/mL, 2 h), and autophagy was evaluated by measuring LC3I and LC3II expression as well as LC3II/I ratio. The upper panel was a representative experiment and the lower panel was the summary of data of LC3II/I expression ratio from 5 separate experiments. GAPDH served as loading control. Data were expressed as normalized ratio of protein band density of LC3II against LC3I, which was normalized with GAPDH

We pre‐treated neurons with different concentrations (1, 5, 10, 25, 50 μmol/L) of propofol for 1 hour, followed by optimal TNF‐α treatment (40 ng/mL, 2 hours) and examined the extent of autophagy. As shown in Figure 2A, we showed that 25 and 50 μmol/L propofol could attenuate TNF‐α‐induced autophagy (*P* < .05 vs TNF‐α treatment). Consistently, 25 and 50 μmol/L propofol greatly attenuated TNF‐α‐modulated LC3I and LC3II expression as well as LC3II/I ratio (Figure 2A, *P* < .05 vs TNF‐α treatment). Please note that lower concentrations (1, 5, 10 μmol/L) of propofol and its solvent 0.1% DMSO had no or only minor effect on TNF‐α‐induced autophagy (Figure 2A,B). Therefore, we ruled out the role of DMSO and inferred that 25 μmol/L propofol may be the minimally effective concentration that inhibited TNF‐α‐induced autophagy in hippocampal neurons.

### 3.3 Propofol inhibited TNF‐α‐induced intracellular calcium accumulation, CAMK II phosphorylation and calpain activation

**Figure 3:**
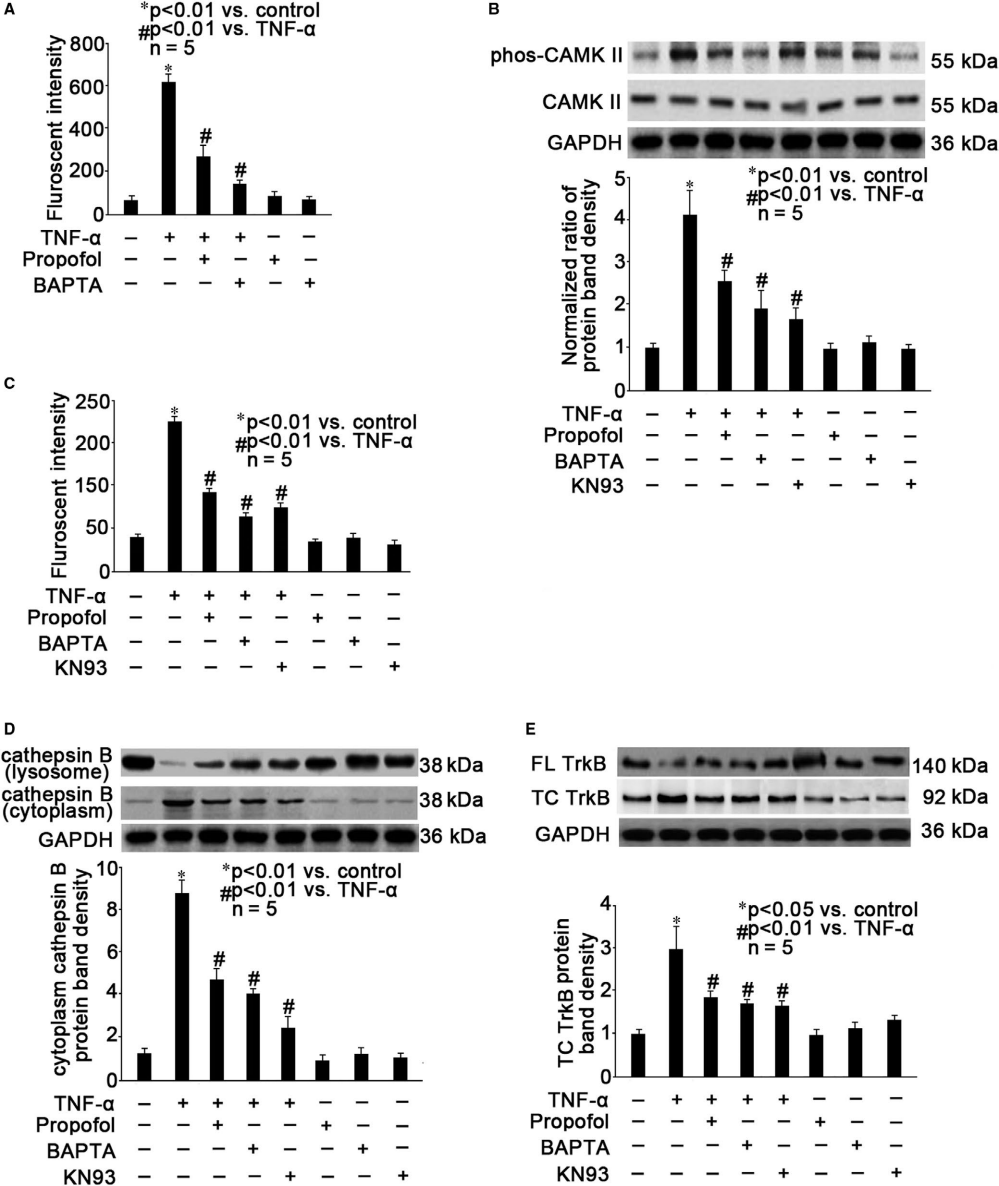
Propofol inhibited TNF‐α‐induced intracellular calcium accumulation, CAMK II phosphorylation, calpain activation, cathepsin B release to cytoplasm and TrkB truncation. Neurons were pre‐treated with 25 μmol/L propofol, 100 μmol/L BAPTA (calcium chelator) or 100 μmol/L KN93 (CAMK II inhibitor), followed by TNF‐α treatment (40 ng/mL, 2h). A, Intracellular calcium was detected by fluorescent assay and the fluorescence was measured by flow cytometry. Data were expressed as fluorescent intensity and were summarized from 5 separate experiments. B, CAMK II expression and phosphorylation were examined by Western blot. The upper panel was a representative experiment and the lower panel was the summary of densitometric data of phosphorylated CAMK II (phos‐CAMK II) from 5 separate experiments. GAPDH served as loading control. Data were expressed as normalized ratio of protein band density of phos‐CAMK II against CAMK II, which was normalized with GAPDH. C, The calpain activity was detected by fluorescent assay with fluorescent reader. Data were expressed as fluorescent intensity and were summarized from 5 separate experiments. D, The expression of cathepsin B in the lysosome and in the cytoplasm was examined by Western blot. The upper panel was a representative experiment and the lower panel was the summary of densitometric data of cathepsin B in the cytoplasm from 5 separate experiments. GAPDH served as loading control. Data were expressed as normalized ratio of protein band density of cytoplasm cathepsin B, which was normalized with GAPDH. E, The expression of full‐length (FL) TrkB and truncated (TC) TrkB was examined by Western blot. The upper panel was a representative experiment and the lower panel was the summary of densitometric data of TC TrkB from 5 separate experiments. GAPDH served as loading control. Data were expressed as normalized ratio of protein band density of TC TrkB, which was normalized with GAPDH

It has been widely accepted that neuronal calcium homoeostasis and calcium‐related molecules such as calpains are closely correlated with neuron autophagy and neurodegenerative diseases.^28^ Accordingly, we examined the effects of propofol and TNF‐α on intracellular calcium and calcium‐dependent signalling pathway. We identified that TNF‐α (40 ng/mL, 2 hours) could increase intracellular calcium concentration (Figure 3A, *P* < .01 vs control neurons), CAMK II phosphorylation (Figure 3B, *P* < .01 vs control neurons) and calpain activation (Figure 3C, *P* < .01 vs control neurons). In addition, we examined the release of lysosomal protease cathepsin B and the truncation of TrkB, which have been proved to be correlated with calpain activation and autophagy.^4^, ^17^, ^18^ We found that TNF‐α induced the release of cathepsin B from lysosome to cytoplasm (Figure 3D, *P* < .01 vs control neurons) and the truncation of TrkB from full‐length TrkB (Figure 3E, *P* < .05 vs control neurons). More importantly, we revealed that all these TNF‐α‐mediated effects were mitigated by 25 μmol/L propofol pre‐treatment (Figure 3A‐E). To confirm the critical role of calcium, neurons were pre‐treated with 100 μmol/L BAPTA (calcium chelator) for 1 hour, followed by TNF‐α treatment (40 ng/mL, 2 hour). We demonstrated that BAPTA could inhibit TNF‐α‐induced intracellular calcium overload (Figure 3A, *P* < .01 vs TNF‐α treatment), CAMKII phosphorylation (Figure 3B, *P* < .01 vs TNF‐α treatment), calpain activation (Figure 3C, *P* < .01 vs TNF‐α treatment) and cathepsin B release to cytoplasm (Figure 3D, *P* < .01 vs TNF‐α treatment) as well as TrkB truncation (Figure 3E, *P* < .01 vs TNF‐α treatment). To confirm the role of CAMK II, we pre‐treated neurons with 100 μmol/L KN93 (CAMK II inhibitor) for 1 hour, followed by TNF‐α treatment (40 ng/mL, 2 hours) and revealed that KN93 inhibited TNF‐α‐modulated CAMKII phosphorylation (Figure 3B, *P* < .01 vs TNF‐α treatment), calpain activation (Figure 3C, *P* < .01 vs TNF‐α treatment) and cathepsin B release to cytoplasm (Figure 3D, *P* < .01 vs TNF‐α treatment) as well as TrkB truncation (Figure 3E, *P* < .01 vs TNF‐α treatment).

### 3.4 Calpain‐2 was involved in TNF‐α‐ and propofol‐modulated calpain activation

**Figure 4:**
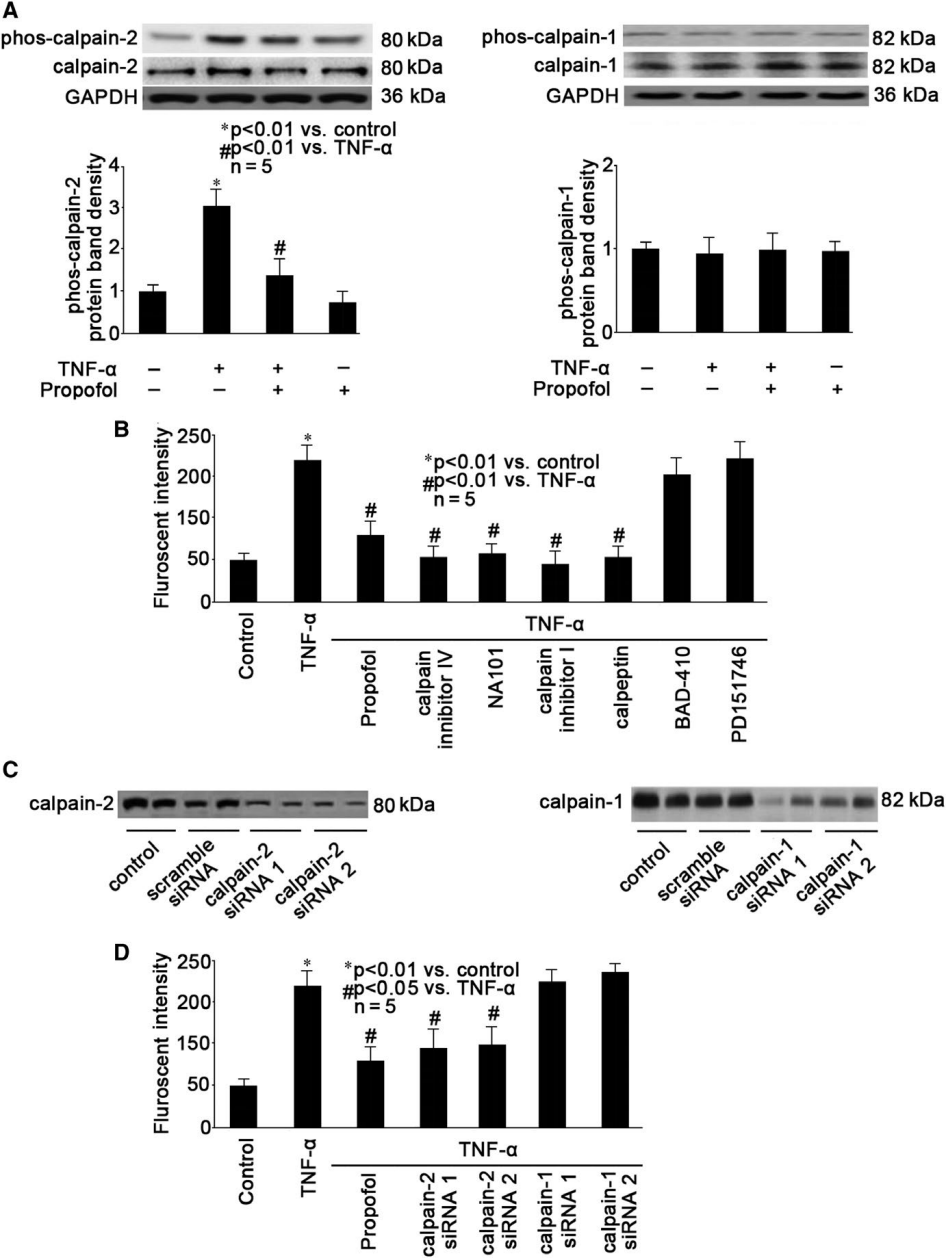
Calpain‐2 was involved in TNF‐α‐ and propofol‐modulated calpain activation. A, Neurons were pre‐treated with 25 μmol/L propofol, followed by TNF‐α treatment (40 ng/mL, 2 h), and the expression as well as phosphorylation of calpian‐1 and calpain‐2 were examined by Western blot. The upper panel was a representative experiment and the lower panel was the summary of densitometric data of phosphorylated calpain‐1 (phos‐calpain‐1, right panel) and phosphorylated calpain‐2 (phos‐calpain‐2, left panel) from 5 separate experiments. GAPDH served as loading control. Data were expressed as normalized ratio of protein band density of s phosphorylated calpain‐1/2 against calpain‐1/2, which was normalized with GAPDH. B, Neurons were pre‐treated with 25 μmol/L propofol, 50 μmol/L calpain inhibitor IV (a calpain‐2 selective inhibitor), 200 nmol/L NA101 (a calpain‐2 selective inhibitor), 20 μmol/L calpain Inhibitor I (a non‐selective calpain inhibitor), 50 μmol/L calpeptin (a non‐selective calpain inhibitor), 20 μmol/L BDA‐410 (a preferential calpain‐1 inhibitor) or 20 μmol/L PD151746 (a preferential calpain‐1 inhibitor), followed by TNF‐α treatment (40 ng/mL, 2 h). The calpain activity was detected by fluorescent assay with fluorescent reader. Data were expressed as fluorescent intensity and were summarized from 5 separate experiments. C, Transfection efficiency of siRNA was evaluated by Western blot. Two calpain‐1 siRNAs (calpain‐1 siRNA1 and calpain‐1 siRNA2) and two calpain‐2 siRNAs (calpain‐2 siRNA1 and calpain‐2 siRNA2) were examined. Untransfected neurons served as control, and scramble siRNA‐transfected neurons served as transfection control. D, Neurons were pre‐treated with 25 μmol/L propofol or transfected with calpain‐1 siRNAs (calpain‐1 siRNA1 and calpain‐1 siRNA2) or calpain‐2 siRNAs (calpain‐2 siRNA1 and calpain‐2 siRNA2), followed by TNF‐α treatment (40 ng/mL, 2 h). The calpain activity was detected by fluorescent assay with fluorescent reader. Data were expressed as fluorescent intensity and were summarized from 5 separate experiments

Calpains constitute a family of enzymes with at least 15 isoforms, among which, calpain‐1 and ‐2 are predominantly expressed in the brain.^17^, ^18^, ^26^, ^27^ We intended to figure out which isoform plays a key role in TNF‐α‐ and propofol‐modulated calpain activation. As shown in Figure 4A, we found that TNF‐α induced the phosphorylation of calpain‐2, which was ameliorated by propofol. While, TNF‐α and propofol had no effect on calpain‐2 expression and calpain‐1 expression as well as phosphorylation (Figure 4A). In addition, we found that TNF‐α‐induced calpain activation was attenuated by pre‐treatment of neurons with 50 μmol/L calpain inhibitor IV (a calpain‐2 selective inhibitor), 200 nmol/L NA101 (a calpain‐2 selective inhibitor), 20 μmol/L calpain Inhibitor I (a non‐selective calpain inhibitor) and 50 μmol/L calpeptin (a non‐selective calpain inhibitor), but not with 20 μmol/L BDA‐410 (a preferential calpain‐1 inhibitor) or 20 μmol/L PD151746 (a preferential calpain‐1 inhibitor) (Figure 4B). The role of calpain‐2 was further confirmed by interfering with the expression of calpain‐1 or calpain‐2 through siRNA transfection. The transfection efficiency was confirmed by Western blot, and as shown in Figure 4C, compared with untransfected control neurons, the expression of calpain‐1 was reduced after transfection of neurons with two different calpain‐1 siRNAs, marked as calpain‐1 siRNA1 and calpain‐1 siRNA2. Also, the expression of calpain‐2 was reduced after transfection of neurons with two different calpain‐2 siRNAs, marked as calpain‐2 siRNA1 and calpain‐2 siRNA2. The transfection of scramble siRNA, serving as transfection control had no effect of calpain expression. More importantly, we reported that in calpain‐2 siRNA‐transfected neurons, TNF‐α only induced calpain activation slightly (Figure 4D). In contrast, calpain‐1 siRNA transfection had no effect on TNF‐α‐modulated calpain activation (Figure 4D).

### 3.5 Propofol‐ mediated effects were mediated through NMDA receptor

**Figure 5:**
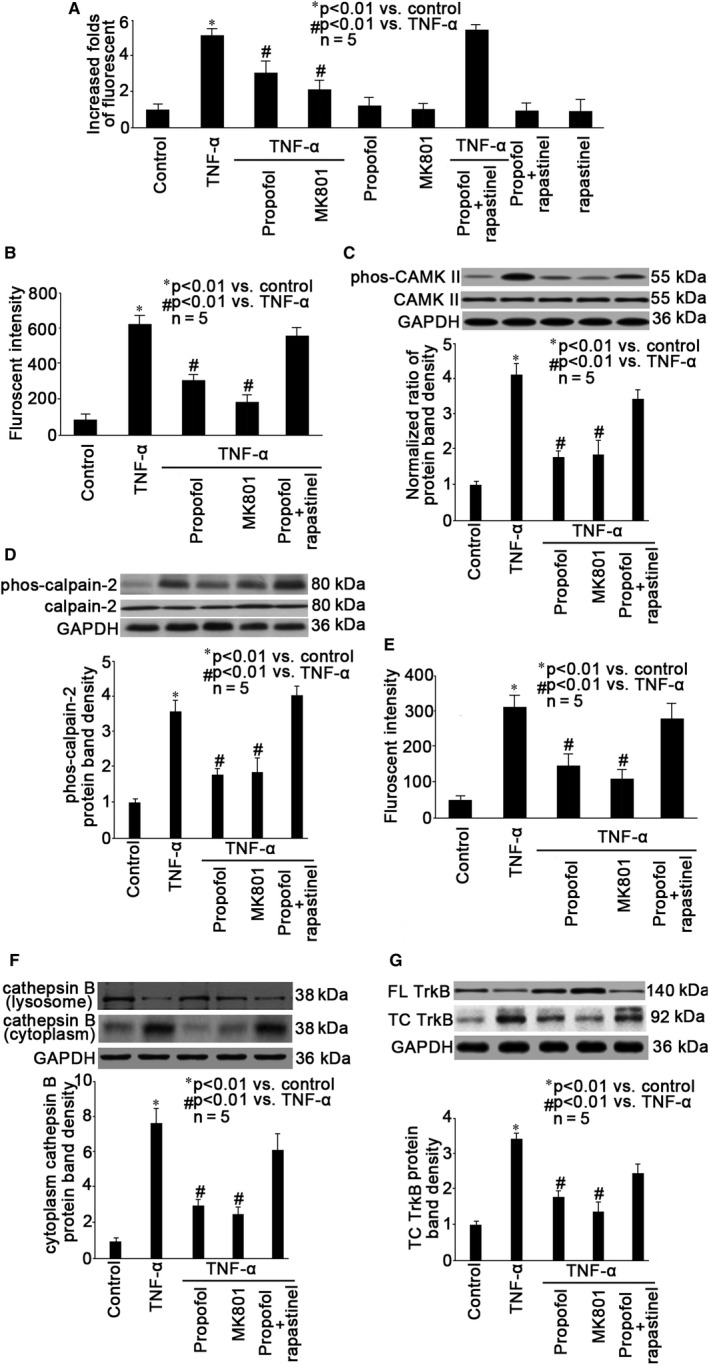
Propofol‐ mediated effects were mediated through NMDA receptor. Neurons were pre‐treated with 25 μmol/L propofol, 10 μmol/L MK801 (a NMDA receptor antagonist), 25 μmol/L propofol plus 20 μmol/L rapastinel (a NMDA receptor activator), followed by TNF‐α treatment (40 ng/mL, 2 h). A, Autophagy was measured by flow cytometry. The fluorescent intensity of each group was recorded. Data were summarized from 5 separate experiments and were presented as increased folds of fluorescent intensity in comparison to control neurons. B, Intracellular calcium was detected by fluorescent assay and the fluorescence was measured by flow cytometry. Data were expressed as fluorescent intensity and were summarized from 5 separate experiments. C, CAMK II expression and phosphorylation were examined by Western blot. The upper panel was a representative experiment and the lower panel was the summary of densitometric data of phosphorylated CAMK II (phos‐CAMK II) from 5 separate experiments. GAPDH served as loading control. Data were expressed as normalized ratio of protein band density of phos‐CAMK II against CAMK II, which was normalized with GAPDH. D, The expression and phosphorylation of calpain‐2 was examined by Western blot. The upper panel was a representative experiment and the lower panel was the summary of densitometric data of phosphorylated calpain‐2 (phos‐calpain‐2) from 5 separate experiments. GAPDH served as loading control. Data were expressed as normalized ratio of protein band density of phos‐calpain‐2 against calpain‐2, which was normalized with GAPDH. E, The calpain activity was detected by fluorescent assay with fluorescent reader. Data were expressed as fluorescent intensity and were summarized from 5 separate experiments. F, The expression of cathepsin B in the lysosome and in the cytoplasm was examined by Western blot. The upper panel was a representative experiment and the lower panel was the summary of densitometric data of cathepsin B in the cytoplasm from 5 separate experiments. GAPDH served as loading control. Data were expressed as normalized ratio of protein band density of cytoplasm cathepsin B, which was normalized with GAPDH. G, The expression of full‐length (FL) TrkB and truncated (TC) TrkB was examined by Western blot. The upper panel was a representative experiment and the lower panel was the summary of densitometric data of TC TrkB from 5 separate experiments. GAPDH served as loading control. Data were expressed as normalized ratio of protein band density of TC TrkB, which was normalized with GAPDH

It is well accepted that the sedative effects of propofol are mainly carried out thought activating gamma‐aminobutyric acid (GABA) receptor, while increasing data indicate the role of NMDA receptor in propofol‐mediated effects in the central nervous system.^23^, ^29^ We examined whether the effects of propofol against TNF‐α‐mediated autophagy was mediated through NMDA receptor. We evaluated autophagy through fluorescent assay, and as shown in Figure 5A, we found that TNF‐α‐induced autophagy was attenuated by pre‐treatment of neurons with 10 μmol/L MK801 (a NMDA receptor antagonist). In contrast, we detected that with the presence of 20 μmol/L rapastinel (a NMDA receptor activator) propofol could not inhibit TNF‐α‐induced autophagy (Figure 5A). In consistence, we reported that MK801 could attenuate TNF‐α‐ induced intracellular calcium accumulation (Figure 5B), phosphorylation of CAMK II (Figure 5C) and calpain‐2 (Figure 5D), calpain activation (Figure 5E) and cathepsin B release (Figure 5F) as well as TrkB truncation (Figure 5G), and the effect of MK801 was similar to that of propofol (Figure 5). We also showed that the effect of propofol on TNF‐α‐ induced intracellular calcium accumulation, phosphorylation of CaMK II and calpain‐2, calpain activation, cathepsin B release and TrkB truncation was counteracted by rapastinel (Figure 5).

## 4 DISCUSSION

In the present study, we examined the effect of pro‐inflammatory cytokine TNF‐α and general anaesthetic agent propofol on autophagy in mouse hippocampal neurons and investigated the underlying mechanism. Our data suggested that propofol may inhibit TNF‐α‐induced autophagy via NMDA receptor and implied the involvement of calcium and calcium‐dependent protease calpain‐2 as a potential mechanism.

### 4.1 The effect of TNF‐α on hippocampal neuron autophagy

Neuroinflammation has been considered as a major pathogenesis of POCD, through exerting noxious effects on neurons, synapsis and BBB.^3^, ^4^, ^5^ Previous studies have revealed that pro‐inflammatory cytokine TNF‐α may induce dopaminergic neuron apoptosis, deteriorate brain microglia and astrocyte function, modulate synaptic plasticity and impair neuronal tight junction as well BBB integrity.^9^, ^10^, ^11^, ^12^, ^13^, ^30^ Hippocampal neurons play important roles in learning and memory, and excessive hippocampal neuron autophagy was revealed to be extensively involved in the development of POCD.^15^, ^31^ Unfortunately, till to now, little is known about whether and how TNF‐α induced hippocampal neuron autophagy.

Autophagy is mainly composed of three types: macroautophagy, microautophagy and chaperone‐mediated autophagy. Macroautophagy is the most common and best studied, and it relies on de novo formation of cytosolic double‐membrane vesicles, autophagosomes, to sequester and transport cargo to the lysosome. Under normal conditions, macroautophagy aids in cellular maintenance by specifically degrading damaged or superfluous organelles, however, excessive macroautophagy and self‐degradation could be deleterious. It was reported that massive neuronal macroautophagy played an important role in the pathogenesis of traumatic brain injury.^32^ In this study, we performed autophagy assay and measured LC3II/ I ratio, which are standard parameters for macroautoghagy assessment. We identified that 40 ng/mL TNF‐α treatment for 2 hours greatly induced autophagy in mouse hippocampal neurons (Figure 1), thus proposing that TNF‐α acts not only as an inflammation mediator but also as a trigger of autophagic impairment and neuronal damage. To date, the principal methods used to monitor autophagic activation are the detection of LC3 processing by Western blot analysis and the detection of autophagosome formation by fluorescence, which were applied in this study. It is noted that autophagy is considered to be a dynamic process that comprises three sequential steps: formation of autophagosomes, the fusion of autophagosomes with lysosomes and degradation. The expression of LC3‐II increased in two cases after autophagy activation. In the first case, autophagy activation increased and autophagic flux was intact. In the second case, autolysosome fusion disorder leads to impaired autophagic flux and accumulation of autophagosomes. We reported that the release of cathepsin B from the lysosomes to cytoplasm significantly increased after TNF‐α treatment, which would affect the fusion of autophagosomes and lysosomes, resulting in impaired autophagic flux (Figure 3D). Accordingly, we believed this is the mechanism highly involved in the TNF‐α‐modulated neuron autophagy. Our finding provided a novel mechanism, through which neuroinflammation may potentially cause POCD.

### 4.2 The role of calcium and calapin‐2 in TNF‐α‐induced hippocampal neuron autophagy

There are multiple signalling pathways involved in autophagy. Rapamycin kinase and target of rapamycin pathway is involved in nutrient and growth factor‐induced autophagy, cAMP‐dependent protein kinase A pathway is activated during glucose‐induced autophagy, adenosine 5′‐monophosphate ‐activated protein kinase plays a role in energy‐mediated autophagy, hypoxia‐inducible factor (HIF) pathway or HIF independent rapamycin kinase pathways may both be activated by hypoxia during autophagy.^33^ In addition, calcium dysregulation is also well accepted as a major intracellular messenger for autophagy.^18^, ^24^, ^28^ It was found that cytosolic calcium and CAMK II was involved in autophagy induced by endoplasmic reticulum (ER) stress and a variety of cellular stressors such as inflammation.^24^ Consistently, we reported that TNF‐α induced intracellular calcium overload and CAMK II activation (Figure 3). Since the TNF‐α‐induced autophagy was inhibited by calcium chelator and CAMK II inhibitor (Figure 3), we believed that calcium at least plays an important role in this event.

Calpains represent a family of calcium‐dependent proteases, which modify the truncation and function of a large number of target proteins, belonging to many classes and including membrane receptors and ion channels, cytoskeletal proteins, protein kinases and phosphatases, transcription factors, as well as regulatory proteins. These proteases have been implicated in numerous cell functions, including cell division, proliferation, migration and death, and their physiological as well as the pathological functions are studied in multiple organs.^18^ Although a plethora of calpain isoforms have been identified, it is recognized that in the brain calpain‐1 and calpain‐2 are the main calpain isoforms and play critical roles in synaptic plasticity, neuron death, neurodegeneration, learning and memory.^17^, ^34^, ^35^ Calpain‐1 (also known as µ‐calpain) and calpain‐2 (also known as m‐calpain) require µmol/L and mmol/L concentrations of calcium for their intracellular activity, respectively. And they play opposite functions in the brain, with calpain‐1 activation being required for triggering synaptic plasticity and neuroprotection, and calpain‐2 limiting the extent of plasticity and being neurodegenerative.^26^, ^27^ In consistent, our data indicated that TNF‐α‐induced hippocampal neuron autophagy was mediated via calpain‐2 activation, since preferential calpain‐2 inhibitors (calpain inhibitor IV and NA101) and calpain‐2 knockdown could attenuate TNF‐α‐induced autophagy, while preferential calpain‐1 inhibitors (BDA‐410 and PD151746) and calpain‐1 knockdown had no such effect (Figure 4B,D). Interestingly, it was demonstrated that calpain‐2 inhibition or knockdown enhanced autophagy and reduced cell death after ischaemia‐reperfusion injury.^36^ Please note that it was performed in liver, and we inferred that the relation between calpain‐2 and autophagy depends on the diversity of stimuli and organs.

Although it has been found that in the epidermal growth factor ‐ and brain‐derived neurotrophic factor (BDNF)‐treated neurons, calpain‐2 could be activated by extracellular signal‐regulated kinase (ERK)‐mediated direct phosphorylation at its serine 50 without increased intracellular calcium concentration.^37^, ^38^ We found that TNF‐α‐induced calpain activation was reduced by calcium chelator and CAMK II inhibitor (Figure 3C), and accordingly, we believed that TNF‐α‐induced calpain‐2 activation was mainly due to calcium signalling rather than ERK.

It was known that calpain‐2 activation may induce autophagy by cleavage of multiple substrates involved in late stages of cell death signalling. These substrates include scaffolding proteins, spectrin, drebrin, phosphate and tension homology deleted on chromsome ten, striatal‐enriched tyrosine phosphatase, glycogen synthase kinase‐3β, cyclin‐dependent kinase, collapsin response mediator proteins, β‐catenin, K‐Cl co‐transporter and metabotropic glutamate receptor.^18^, ^25^, ^27^ Calpain‐2 also induces the truncation of full‐length TrkB,^26^ through which BDNF plays an important role in neuronal plasticity, learning and memory. Dysregulation of BDNF/TrkB signalling is correlated with neuron autophagy^26^ and POCD.^4^ In addition, calpain‐2 may cause the truncation of carbonylated heat shock protein 70, leading to the destabilization of lysosomal membranes and the release of cathepsins B,^26^ which is closely correlated with autophagy. In this study, we demonstrated that TNF‐α induced lysosomal cathepsin B release and TrkB truncation (Figure 3). Our data implied that calpain‐2‐mediated autophagy proceeded at least through cathepsin B release and TrkB truncation.

### 4.3 Propofol inhibited TNF‐α‐induced neuron autophagy via NMDA receptor and calcium pathway

Propofol is a most widely used intravenous anaesthetic, and the action of propofol involves a positive modulation of the inhibitory function of the neurotransmitter g‐aminobutyric acid through GABA receptors. Meanwhile, other receptors, such as glycine receptor, nicotinic receptor and M1 muscarinic receptor have been proposed to play roles in propofol‐mediated pleiotropic effects.^39^ Recently, the role of NMDA receptor in propofol‐mediated effects has been suggested. NMDA receptor belongs to the family of ionotropic glutamate receptor, which mediates most excitatory synaptic transmission in mammalian brains. Activation of NMDA receptors results in the opening of an ion channel which is non‐selective to cations, including sodium, potassium and calcium. Calcium permeation triggered by activation of NMDA receptors is the pivotal event for initiation of neuronal plasticity. In this study, we showed that propofol and NMDA receptor antagonist could inhibit calcium overload (Figure 5B), implying the role of NMDA receptor in modulating intracellular calcium imbalance. In addition, NMDA receptor and calcium signalling have been suggested to be involved in propofol‐mediated anti‐inflammatory effects in BV2 microglia.^23^ Propofol may also disrupt aerobic glycolysis in colorectal cancer cells and attenuate pancreatic cancer malignant via inactivation of NMDA receptor and reducing calcium overload.^40^, ^41^ In addition, propofol was found to attenuate the adhesion of tumour and endothelial cells via inhibiting NMDA receptor.^42^ In the present study, we reported that propofol reduced TNF‐α‐induced calcium overload, calcium‐related pathway and autophagy through blocking NMDA receptor. Previous studies suggested that NMDA receptor antagonist may selectively block the function of extrasynaptic NMDA receptors, thus serving as a disease‐modifying therapeutic strategy for Alzheimer's disease.^43^, ^44^ Similarly, we inferred that NMDA antagonist may be a therapeutic strategy for POCD. We reported currently that propofol may attenuate TNF‐α‐induced autophagy in mouse hippocampal neurons, and we have proved previously that propofol may protect against TNF‐α‐modulated apoptosis and cell viability in mouse hippocampal neurons (9). Combining these data, we further believed the protective effect of propofol against inflammation‐mediated neuron damage.

### 4.4 Limitations

We realized there are several limitations within this study. Firstly, a major distinction between calpain‐1 and calpain‐2 is the level of calcium required for activation. In in vitro* *assays, calpain‐2 has been shown to require 400‐800 μmol/L calcium, while calpain‐1 has a lower requirement of 3‐50 μmol/L calcium. In the present study, we only measured intracellular calcium overload by fluorescent assay without measuring the exact intracellular calcium concentration. Secondly, calpain could be activated by multiple mechanisms, such as phosphorylation, translocation and cleavage. In addition, calpain activity could be regulated by a specific endogenous inhibitor calpastain, which is expressed throughout the brain. In this study, we only measured the phosphorylation of calpain‐1 and calpain‐2. Further investigation is needed to examine other potential mechanisms for modulating calpain activity. Thirdly, under physiological conditions, the cytosolic calcium concentration is generally estimated to be 0.1‐0.3 μmol/L, and extracellular calcium concentration is approximately 2 mmol/L. In response to stimuli, calcium could be either released from intracellular stores, such as ER and mitochondria, or could cross plasma membranes through ionotropic receptors and voltage‐gated calcium channels. Here in this study, we did not examine the source of TNF‐α‐induced intracellular calcium overload, which is of interest and may be investigated in the future.

## 5 CONCLUSIONS

Propofol, via NMDA receptor, inhibited TNF‐α‐mediated hippocampal neuron autophagy. The mechanism may involve calcium and calcium‐dependent signalling pathway, especially CAMK II and calpain‐2. Our findings may provide a novel therapeutic target for inflammation‐induced neuron death and neurodegeneration.

## CONFLICTS OF INTEREST

The authors confirm that there are no conflicts of interest.

## AUTHOR CONTRIBUTION

**Ying Li:** Resources (equal); Writing‐original draft (equal). **Zhiyong He:** Resources (equal); Writing‐original draft (equal). **Hu Lv:** Validation (equal). **Wei Chen:** Supervision (equal); Writing‐original draft (equal); Writing‐review & editing (equal). **Jiawei Chen:** Resources (equal); Supervision (equal); Writing‐original draft (equal); Writing‐review & editing (equal).

## Data Availability

The data that support the findings of this study are available from the corresponding author upon reasonable request.
